# Development of High-Fidelity Automotive LiDAR Sensor Model with Standardized Interfaces

**DOI:** 10.3390/s22197556

**Published:** 2022-10-05

**Authors:** Arsalan Haider, Marcell Pigniczki, Michael H. Köhler, Maximilian Fink, Michael Schardt, Yannik Cichy, Thomas Zeh, Lukas Haas, Tim Poguntke, Martin Jakobi, Alexander W. Koch

**Affiliations:** 1 IFM—Institute for Advanced Driver Assistance Systems and Connected Mobility, Kempten University of Applied Sciences, Junkersstrasse 1A, 87734 Benningen, Germany; 2 Institute for Measurement Systems and Sensor Technology, Technical University of Munich, Theresienstr. 90, 80333 Munich, Germany; 3 Blickfeld GmbH, Barthstr. 12, 80339 Munich, Germany; 4 IPG Automotive GmbH, Bannwaldallee 60, 76185 Karlsruhe, Germany

**Keywords:** advanced driver-assistance systems, automotive LiDAR sensor, open standard, standardized interfaces, open simulation interface, functional mock-up interface, functional mock-up unit, co-simulation environment, CarMaker, silicon photomultipliers detector, time domain signal, point clouds, proving ground

## Abstract

This work introduces a process to develop a tool-independent, high-fidelity, ray tracing-based light detection and ranging (LiDAR) model. This virtual LiDAR sensor includes accurate modeling of the scan pattern and a complete signal processing toolchain of a LiDAR sensor. It is developed as a functional mock-up unit (FMU) by using the standardized open simulation interface (OSI) 3.0.2, and functional mock-up interface (FMI) 2.0. Subsequently, it was integrated into two commercial software virtual environment frameworks to demonstrate its exchangeability. Furthermore, the accuracy of the LiDAR sensor model is validated by comparing the simulation and real measurement data on the time domain and on the point cloud level. The validation results show that the mean absolute percentage error (MAPE) of simulated and measured time domain signal amplitude is 1.7%. In addition, the MAPE of the number of points $N_{points}$ and mean intensity $I_{mean}$ values received from the virtual and real targets are 8.5% and 9.3%, respectively. To the author’s knowledge, these are the smallest errors reported for the number of received points $N_{points}$ and mean intensity $I_{mean}$ values up until now. Moreover, the distance error $d_{error}$ is below the range accuracy of the actual LiDAR sensor, which is 2 cm for this use case. In addition, the proving ground measurement results are compared with the state-of-the-art LiDAR model provided by commercial software and the proposed LiDAR model to measure the presented model fidelity. The results show that the complete signal processing steps and imperfections of real LiDAR sensors need to be considered in the virtual LiDAR to obtain simulation results close to the actual sensor. Such considerable imperfections are optical losses, inherent detector effects, effects generated by the electrical amplification, and noise produced by the sunlight.

## 1. Introduction

**Figure 1:**
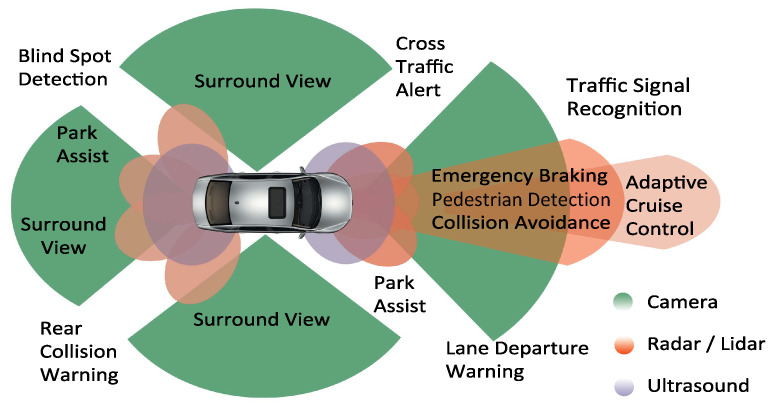
ADAS functions used in modern vehicles, Source: adapted from [2].

**Figure 2:**
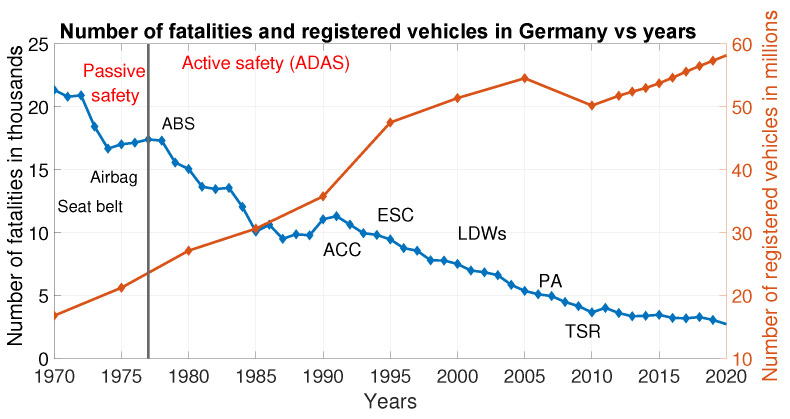
Decrease in road fatalities despite the increase in the number of motor vehicles due to the advances of ADAS in Germany, Source: adapted from [1,3]. ABS, anti-lock braking system; ACC, adaptive cruise control; ESC, electronic stability control; LDW, lane departure warning; PA, parking assistant; TSR, traffic sign recognition.

Advanced driver-assistance systems (ADAS) are currently an area of focus in the automotive industry. Modern vehicles are equipped with different ADAS, increasing the driver’s comfort and safety, as depicted in Figure 1. According to the German Federal Statistical Office, fatalities in road accidents in Germany dropped from 21,330 in 1970 to 2562 by 2021 [1], regardless of the tremendous increase in the number of motor vehicles that took place during these years. Figure 2 summarizes the ADAS role in reducing the number of fatalities in traffic accidents.

The complexity of ADAS is also increasing rapidly, and validation of such systems is becoming challenging. The validation of such a complex system in the real world is expensive and time-consuming. According to the RAND cooperation statistical studies, 5 billion km of test driving is required to demonstrate the autonomous vehicle failure rate being lower than that of humans [4]. Similar statistical consideration given in [5] also proves that 240 million km of the test drive is required for the verification of the ADAS, which is not feasible to attain. Numerous validation processes have been adopted to overcome this challenge, model-in-the-loop (MiL), hardware-in-the-loop (HiL), and software-in-the-loop (SiL) [5]. Moreover, we see increasing efforts and work from academia and industry with regard to virtual validation processes. Research projects including VIVID [6], DIVP [7], VVM [8], and SET level [9] also endorsed the same concept. In addition, the automotive industry has started considering type approval based on virtual tests [10]. In addition, the ADAS complexity requires joint efforts and collaboration of industrial players from different domains, which is only possible with an effective exchange of models without intellectual property (IP) violation [11]. Therefore, open standards and interfaces have been introduced, including an open simulation interface (OSI) and functional mock-up interface (FMI) [11,12].

The virtual environment and environmental perception sensors exhibit the complexity and behavior of real-world scenarios, and sensors are essential elements and enablers in all these activities [13]. However, the state-of-the-art virtual scenarios and environmental perception sensors provided by simulation tool vendors typically offer a generic, parameterizable model of the optical or electromagnetic wave propagation but often do not consider sensor-specific effects in detail. Moreover, no commonly accepted metrics or standards exist to prove the fidelity of virtually developed environmental perception sensor models and scenarios [14].

This paper contributes to the ADAS virtual testing and validation with a tool-independent, high-fidelity LiDAR sensor model. The proposed model is developed by using a standardized OSI 3.0.2 and FMI 2.0. It was integrated successfully into the virtual environment of CarMaker from IPG Automotive, and the AURELION of dSPACE to verify the exchangeability [15,16]. The operational performance of the LiDAR FMU model is the same, irrespective of the tool used. The presented virtual sensor includes the complete signal processing toolchain of the Blickfeld Cube 1 LiDAR sensor. Moreover, it also considers the optical, electrical, and environmental effects to generate a realistic output. Furthermore, a real-world static test scenario is accurately constructed in the virtual environment of CarMaker. Finally, real and virtual test results are compared to verify the fidelity of the LiDAR sensor model on the time domain and point cloud levels. Furthermore, key performance indicators (KPIs) are defined to authenticate the accuracy of the sensor model at the point cloud level.

The paper is structured as follows: Section 2 describes the LiDAR sensor background. Then, an overview of the state-of-the-art LiDAR sensor models is given in Section 3. Section 4 describes the modeling approach of the proposed LiDAR sensor model. The LiDAR FMU modules specifications are explained in Section 5. The results are discussed in Section 6. Finally, Section 7 and Section 8 provide the conclusion and outlook.

## 2. Background

LiDAR is a range measurement technique that has been used in the military and aviation fields for many decades. However, since the first realization of an autonomous vehicle on the road, original equipment manufacturers (OEMs) have enhanced vehicles’ autonomous capabilities by installing different ADAS [17]. As a result, LiDAR technology has become indispensable for autonomous driving (AD) due to its better angular resolution, and field-of-view (FoV) than RADAR [18].

### LiDAR Working Principle

**Figure 3:**
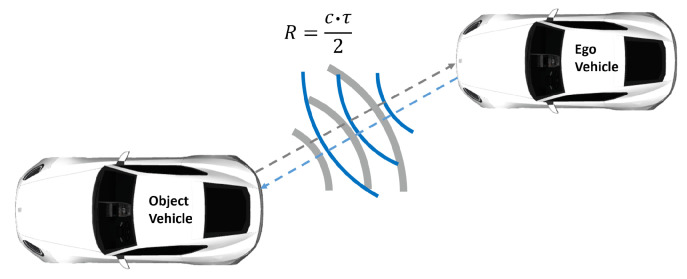
LiDAR working principle. LiDAR sensor mounted on ego vehicle simultaneously sends and receives the laser light partly reflected from the surface of the target and measures the distance.

The LiDAR sensor measures the round-trip delay time (RTDT) that laser light takes to hit an object and returns to calculate the range [19], as depicted in Figure 3.

We have

(1) $$R=\frac{c\cdot \tau }{2},$$

where *R* denotes the target range, *c* is the speed of light, and τ is the RTDT, also known as time of flight (ToF). The LiDAR sensor measures the range and, together with the spatial laser beam deflection, the position by using pulsed or modulated waveforms [19].

## 3. State of the Art

Automotive perception sensor models can be divided into three categories; ideal, phenomenological, and physical models depending on their modeling approach and covered effects [20].

Ideal sensor models, also known as “ground truth (Ground truth provides the simulated objects’ actual values, dimensions, position, velocities, orientation, and bounding box.)” sensor models, use the object list provided by the simulation framework in the world coordinate system as an input. The term ground truth is borrowed from remote sensing, where it refers to information collected on location for data calibration [21]. These models’ output is a filtered object list as per the sensor-specific FoV [22]. The ideal LiDAR sensor models are presented in [23,24], and they do not consider any sensor-related imperfections except FoV and object occlusion. Therefore, these models have low complexity, require less computation time, and can test the highly automated driving (HAD) function operation in the early stage of development. It should be noted that the ideal models which are described in the literature, are mostly generic, and they can fulfill the requirements of different environmental perception sensor types, including LiDAR, RADAR, and camera [23,24]. OSI provides the *osi3::GroundTruth* interface for such models.

Phenomenological sensor models use the object list as an input and apply weather conditions, false alarms (positive/negative), detection probability, and sensor-related effects, including FoV and limited detection range. This type of sensor model output either raw data (point clouds) for LiDAR sensors or object lists [25]. Muckenhuber et al. [26] proposed a generic sensor model that requires a ground truth object list as an input. The model’s output is a sensor-specific object list, including FoV, object class definition, occlusion, and probability of false positive and false negative detections. Linnhoff et al. [27] introduced an object-based LiDAR sensor model that outputs object lists and considers partial occlusion of objects, limitation of angular view, and decreases in the effective range due to atmospheric attenuation. Hirsenkorn et al. [25] presented a generic non-parametric statistical methodology to replicate the behavior of the real-world sensor. The developed model includes various sensor errors that include ranging, latency, false-positive, and occlusion. The model’s output can be either an object list or raw data. A LiDAR model consisting of geometrical and physical submodules is presented in [28,29]. The input of the geometrical model is the object list, including the occlusion, FoV, and beam divergence. The model’s output is an object list that can be extended to point clouds.

Physical sensor models are based on the physical aspects and are numerically complex. Hence, they require a lot of computational power and, thus, might not be real-time capable. The subsequent models use the rendering techniques provided by the simulation framework as input and generate the raw data (point clouds) as an output containing distance, intensity, and timestamp. Several rendering techniques generate the synthetic LiDAR sensor raw data; ray tracing, ray casting, rasterization (z-buffers), and ray path [14,30]. Philipp et al. [31] developed a ray casting-based LiDAR sensor model to generate point clouds. Their presented model includes beam divergence, signal-to-noise ratio (SNR), detection threshold, and material surface reflection properties. Gschwandtner et al. [32] introduced the Blender sensor simulation (Blensor), an open-source LiDAR plugin. The Blensor toolkit uses the ray casting mechanism of Blender, and it considers the sensor noise, materials’ physical properties, and free space path losses (FSPL). Goodin et al. [33] established a ray casting LiDAR sensor model and incorporated it into the virtual navigation environment (VANE). The model can simulate the effects of beam divergence and a Gaussian beam profile. In [34], an open-source multi-purpose LiDAR simulator HELIOS is proposed. HELIOS exploits a ray casting approach and provides the scan pattern of four beam deflection units: fiber array, rotating, oscillating, and conic mirror. The effects of beam divergence, atmospheric attenuation, scanner efficiency, and material surface properties are also modeled. Hanke et al. [35] apply a ray tracing rendering technique for generating synthetic point clouds. Moreover, the suggested model includes beam divergence, material reflection properties, detection threshold, noise effects, and atmospheric attenuation. Li et al. [28] developed a physical sensor model that requires ray tracing data as an input. In addition, the model contains beam divergence and power loss due to rain, fog, snow, and haze. Zhao et al. [29] extend the work of [28]. Their model takes the probability of false alarms due to the backscattering from water droplets. In addition, the physical effects of beam divergence and object surface reflectivity were studied by Goodin et al. [36], who also analyzed the LiDAR signal attenuation and range error due to the rainfall. The commercial and open-source simulation platforms also provide the LiDAR sensor models with different fidelity levels.

CARLA is an open-source simulation environment that offers a LiDAR model that simulates laser rays by using ray casting. The CARLA LiDAR model takes into consideration physical effects, noise, the drop-off in intensity, and the number of point clouds due to external perturbations [37]. CarMaker provides the real-time capable ray tracing-based LiDAR model known as LiDAR raw signal interface (LiDAR RSI). The LiDAR RSI regards propagation losses, object geometry, material surface properties, and incidence angle of ray for intensity calculation of point clouds [20]. DYNA4 from Vector offers a LiDAR model which uses a ray casting technique that outputs raw data intensity based on physical effects, the material surface reflectivity, and ray angle of incidence [38]. The VTD from Vires LiDAR model operates on a ray tracing engine from NVIDIA [14,35]. The AURELION LiDAR model is also based on ray tracing. It considers material surface reflectivity, covers noise, atmospheric attenuation, and fast motion scan effect [39].

This work categorizes the ideal and phenomenological sensor models as low fidelity because of their simplified modeling approach and covered effects. For instance, the abovementioned physical sensor models simulate point clouds according to ray tracing or ray casting detections but don’t consider sensor-specific effects in detail. Such considerable effects are optical losses, inherent detector effects, effects generated by the electrical amplification, and noise produced by sunlight. That is why we classified them as medium-fidelity LiDAR sensor models. On the other hand, the sensor model proposed in this work considers ray tracing detection as input and applies sensor-specific imperfections of the LiDAR sensor to output the realistic time domain and point cloud data. Therefore, the sensor model considers the complete signal processing steps of an actual LiDAR sensor. Because of that, the proposed LiDAR model is classified as a high-fidelity sensor model.

The imperfections implemented in the proposed high-fidelity model significantly impact LiDAR sensor performance. To prove this point, we have compared the presented LiDAR model, the state-of-the-art LiDAR model from commercial software results, with the real measurements at the point cloud level. In addition, the presented LiDAR sensor model is also validated at the time domain level. An overview of the working principle, covered effects, and validation approaches of the LiDAR sensor models presented in this section is listed in Table 1.

## 4. LiDAR Modeling Building Blocks

The modeling and simulation of ADAS sensors are complex, multi-domain, concurrent, and distributed, requiring expert teams from different disciplines to develop and authenticate. However, the rapid development of such a system is possible if every participant prepares their partial solution and integrates it with other partners’ solutions with ease [41].

### 4.1. Open Standards

As given in Section 1, the open standards gained significant interest from the automotive industry in the last few years because it allows the accessible exchange of simulation models between different tools. The LiDAR model developed in this work is for industrial use. Therefore, we used standardized interfaces FMI and OSI to make it tool-independent. We have verified the interchangeability of the proposed sensor model by successfully integrating it into the co-simulation environment of CarMaker and AURELION.

The co-simulation framework provides flexibility to a couple more than one simulation model by using standardized interfaces FMI and OSI [41].

### 4.2. Functional Mock-Up Interface

As mentioned earlier, generic, standardized interfaces are required in the automotive industry to exchange the model between different simulation tools without IP infringement [41]. Therefore, FMI is a solution to this industrial need. It was an initiative of Daimler AG with the primary aim to improve the exchange of simulation models between the OEMs and suppliers [11].

The FMI standard 2.0 contains two types of protocols [42]:

FMI for Model Exchange is an interface to the dynamic system model described by differential, algebraic and discrete-time equations. Dynamics models don’t have a solver, and their C-code is generated by simulation environments that other simulation tools can also use in online and offline simulations.

FMI for Co-Simulation is an interface to connect more than one simulation tool and subsystems in a co-simulation environment. Each subsystem in the co-simulation interface has its own solver to solve independently between two communication steps. The master algorithm steers the data sharing between the subsystems (slaves).

**Figure 4:**
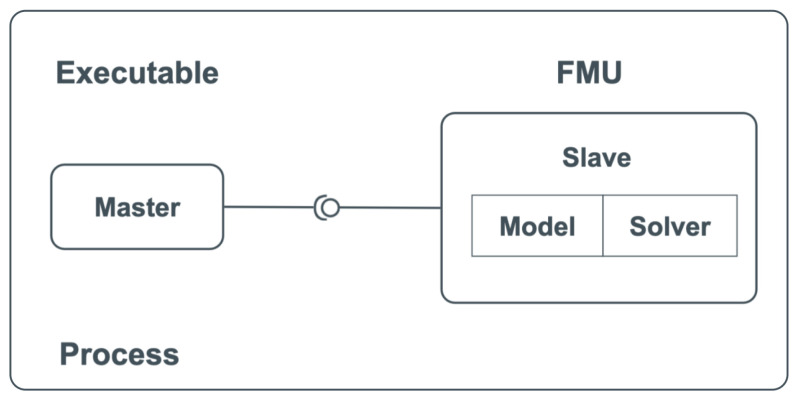
Co-simulation stand-alone use case [11].

The focus of this research paper is on FMI for a co-simulation stand-alone use case. FMI co-simulation stand-alone can be applied in different use cases as given in [42], but this paper focuses on the single process use case shown in Figure 4. In such a case, master and slave both run on the same process. The master controls the coupled simulation, and the slaves consist of the model and solver. FMU is the component that implements the FMI interface [11].

### 4.3. Open Simulation Interface

As mentioned earlier, virtual validation of ADAS and AD is indispensable. Therefore, logical interfaces from the virtual sensors to ADAS are also required. OSI is the first reference implementation of the ISO 23150 for the virtual development and validation of ADAS systems and sensors endorsed in the Pegasus Project [43]. OSI is a generic interface that uses a protocol buffer message format developed by Google to exchange information between the environmental simulation tools, ADAS sensor models, and ADAS [12]. It also provides the flexibility to integrate the sensor models into the co-simulation environment by using the so-called OSI sensor model packaging (OSMP) [44]. The OSMP FMUs transfer the positions and the sizes of the Google protobufs to exchange the data between the simulation environment and sensor model [45]. To study the detailed description of the OSI interfaces, the reader is referred to [12].

## 5. LiDAR Sensor Model

**Figure 5:**
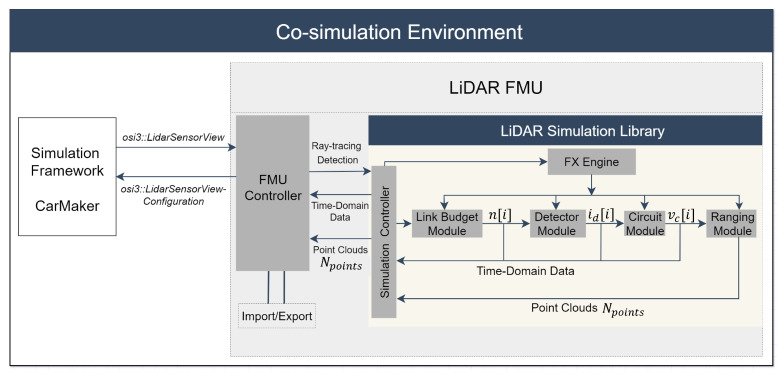
Co-simulation framework of the LiDAR FMU model.

Figure 5 depicts the toolchain and the signal processing steps of the proposed LiDAR model. The sensor model considers the scan pattern and complete signal processing steps of Blickfeld Cube 1. As mentioned earlier in Section 1, the model itself is built as an OSMP FMU and uses the virtual environment of CarMaker. It provides the ray tracing framework with a bidirectional reflectance distribution function (BRDF) that considers the direction of the incident ray θ, material surface, and color properties [46]. The LiDAR FMU model uses the ray tracing module of CarMaker. The material properties of the simulated objects, angle-dependent spectral reflectance $R_{\lambda }\left(\theta \right)$, and reflection types, including diffuse, specular, retroreflective, and transmissive, are specified in the material library of CarMaker.

The FMU controller passes the required input configuration to the simulation framework via *osi3::LidarSensorViewConfiguration*. The simulation tool verifies the input configuration and provides the ray tracing detections via *osi3::LidarSensorView::reflection* interface time delay τ and relative power $P_{rel}\left(R\right)$ [45].

Afterward, the FMU controller calls the LiDAR simulation library and passes the ray tracing data for further processing. The central component of the simulation library is the simulation controller. It is used as the primary interface component to provide interactions with the library, for instance, configuring the simulation pipeline, inserting ray tracing data, executing the simulation’s steps, and retrieving the results.

The next block in the pipeline is the link budget module, which calculates the photons over time. The task of the detector module is to capture these photons’ arrivals and convert them into an electrical current signal $i_{d}\left[i\right]$. In the proposed LiDAR model, we have implemented silicon photomultipliers (SiPM) as a detector [47]. Still, it can also support avalanche photodiode (APD) and single-photon avalanche diode (SPAD) detector models.

The third block in the pipeline is the circuit module. Its task is to amplify and convert the detector’s photo current signal $i_{d}\left[i\right]$ to a voltage signal $v_{c}\left[i\right]$ that is processed by the ranging module.

The last part of the toolchain is the ranging module, which determines the range and intensity of the target based on the $v_{c}\left[i\right]$ received from the analog circuit for every reflected scan point. Finally, the effect engine (FX engine) is a series of interfaces that interacts with environmental or sensor-related effects and the blocks of the simulation pipeline. These interactions can involve, for example, the consideration of thermal noise in electrical components, signal attenuation due to weather phenomena, and backscattering. It should be noted that this paper only considers the environmental condition sunlight effect.

This section will cover a detailed description of scan patterns and LiDAR simulation library components.

### 5.1. Scan Pattern

**Figure 6:**
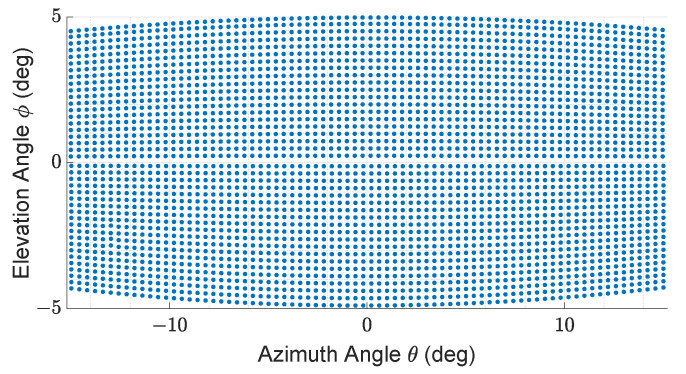
Specification of scan pattern used by LiDAR FMU model and LiDAR sensor: $30^{\circ }$ horizontal and $10^{\circ }$ vertical FoV, 80 scan lines, frame mode only up, $0.4^{\circ }$ horizontal angle spacing, frame rate 6.7 Hz, maximum detection range is 250 m, and minimum detection range is 4.80 m.

**Figure 7:**
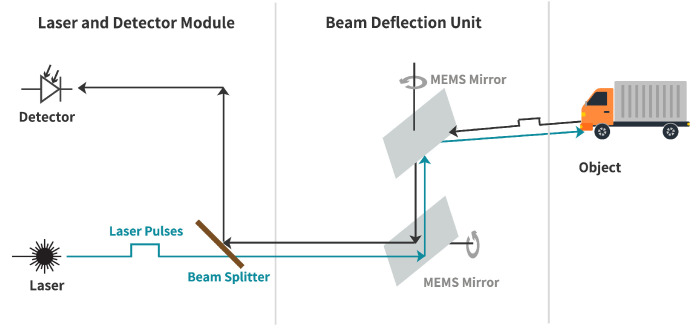
Block diagram of MEMS LiDAR sensor. Source: adapted from [49].

The described LiDAR FMU model uses the Blickfeld Cube 1 elliptical shape scan pattern as given in Figure 6. Cube 1 is comprises of a single laser source to emit laser pulses and a beam deflection unit, a so-called scanner that deflects the beam to obtain an environment image. The scanner deflects the laser beam using two 1D microelectromechanical mirrors (MEMS) scanners oriented horizontally and vertically, and having a phase difference of $45^{\circ }$ [48]. The block diagram of the MEMS LiDAR sensor is shown in Figure 7. The scan pattern is imported inside the LiDAR FMU via the FMU Controller.

### 5.2. Link Budget Module

The relative power $P_{rel}\left(R\right)$ obtained from the ray tracing module does not consider the environmental condition of sunlight and optical losses. The link budget module considers these effects, and the received power $P_{rx}\left(t\right)$ can be given as

(2) $$P_{rx}\left(t\right)=\underset{P_{rel}\left(R\right)}{\underbrace{\rho \frac{d_{aperture}^{2}}{4R_{trg}^{2}}cos\left(\theta \right)}}T_{atm}^{2}T_{opt}P_{tx}\left(t\right),$$

where ρ is the target reflectivity, $d_{aperture}$ denotes the diameter of the optical aperture, $R_{trg}$ is the target range, the direction of the incident ray is given by θ, receiver optics loss factor is given by $T_{opt}$, $T_{atm}$ shows the atmospheric loss factor, and the transmit power is denoted by $P_{tx}\left(t\right)$ [50].

The total received power $P_{tot}\left(t\right)$ by the detector over time can originate from different sources including internal reflection $P_{int}\left(t\right)$, target receive power $P_{rx}\left(t\right)$, and sunlight power $P_{sun}$. That is why $P_{tot}\left(t\right)$ can be given as

(3) $$P_{tot}\left(t\right)=P_{int}\left(t\right)+P_{rx}\left(t\right)+P_{sun}.$$

The $P_{sun}$ can be calculated as

(4) $$P_{sun}=\frac{\rho d_{aperture}^{2}T_{opt}T_{atm}}{4R_{trg}^{2}}\int_{A_{trg}}\int_{BW_{opt}}I_{\lambda ,A}d\lambda dA,$$

where the illuminated area of the laser spot on the target is $A_{trg}$, $BW_{opt}$ denotes the optical bandwidth of the bandpass daylight filter, the optical loss factor is given by $T_{opt}$, $R_{trg}$ is the target distance, and $I_{\lambda ,A}$$\left(\frac{W}{m^{2}\cdot nm}\right)$ is the solar spectral irradiance at air mass 1.5 [50]. In this work, $I_{\lambda ,A}$ values are taken from the ASTM G173-03 standard [51].

It is possible to model the optics at the photon level based on the power equation to make the simulation more accurate. For this approach, the power signal must be sampled with time interval of Δt [47]. Then, the sampled power equation takes the form of

(5) $$P_{tot}\left[i\right]=P_{int}\left[i\right]+P_{rx}\left[i\right]+P_{sun},$$

with t=i·Δt. The mean of incident photons $\bar{n}\left[i\right]$ on the SiPM detector within a one-time bin can be written as

(6) $$\bar{n}\left[i\right]=\frac{P_{tot}\left[i\right]\cdot \Delta t}{E_{ph}},$$

where $E_{ph}=h\nu$ is the energy of a single laser photon at the laser’s wavelength, *h* is the Planck constant and ν is the frequency of the photon [52]. The SiPM detector generates Poisson distributed shot noise due to the statistical arrival of photons. That is why the arrival of photons can be modeled as a Poisson process [53]

(7) $$n\left[i\right]=P(\bar{n}\left[i\right]).$$

**Figure 8:**
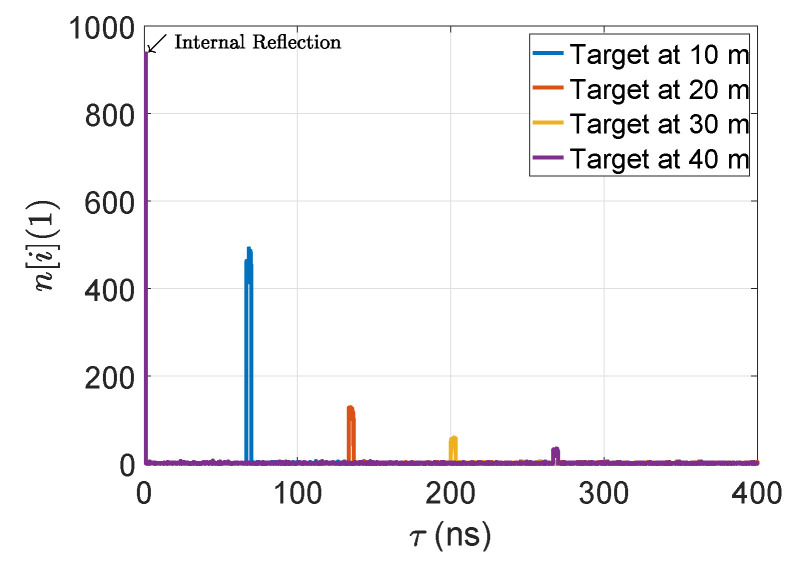
The output of the implemented link budget module for 5% reflective point scatter targets.

The output of the link budget module is given in Figure 8.

### 5.3. SiPM Detector Module

We have implemented the SiPM detector module that provides an output current proportional to the number of photons [47]. In contrast to the SPAD, the SiPM detector yields better multi-photon detection sensitivity, photon number resolution, and extended dynamic range [54,55]. The SiPM detector response for a given photon signal can be calculated as

(8) $$i_{d}\left[i\right]=S_{i}\cdot \left(h_{SiPM}\left[i\right]*n\left[i\right]\right),$$

where $S_{i}$ is the SiPM detector sensitivity, the impulse response of the detector is given as $h_{SiPM}$. $S_{i}$ is given as

(9) $$S_{i}=1-e^{\left(-t/\tau_{delay}\right)},$$

$\tau_{delay}$ is the SiPM recovery time [47,54,55].

**Figure 9:**
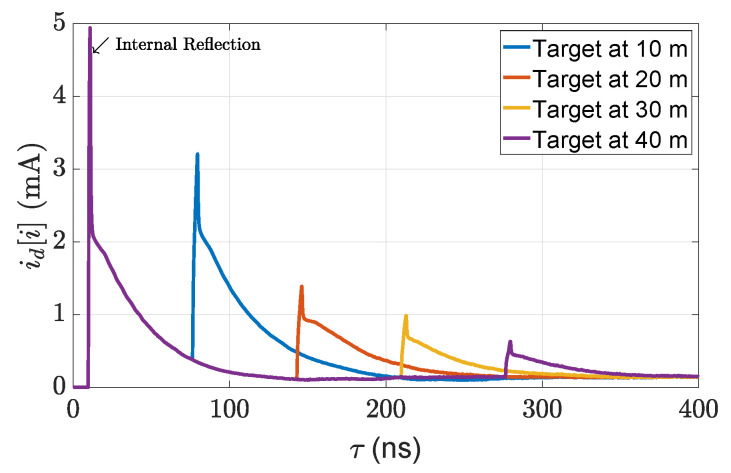
The output of the implemented SiPM detector module for 5% reflective point scatter targets.

The output of the SiPM detector module is given in Figure 9.

### 5.4. Circuit Module

**Figure 10:**
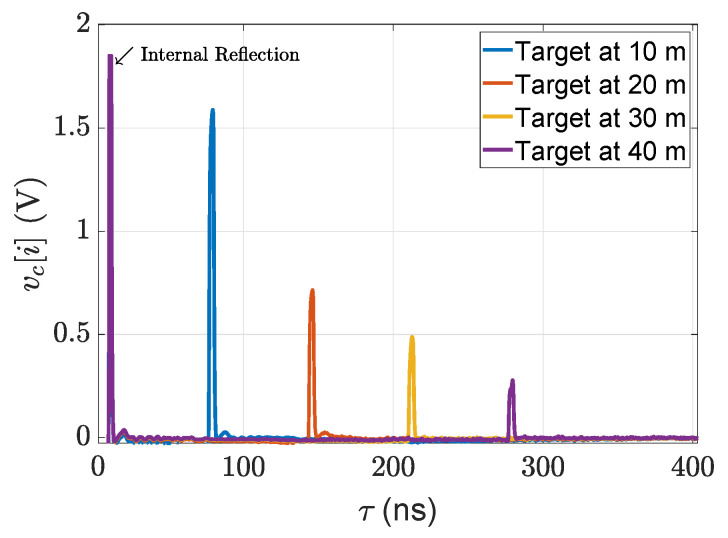
The output of the circuit module for 5% reflective point scatter targets.

We use the small-signal transfer function H(f) of the analog circuit model to obtain the voltage signal $v_{c}\left[i\right]$,

(10) $$v_{c}\left[i\right]=v_{c0}+\Delta v_{c}\left[i\right]=v_{c0}+F^{-1}\left\{H\left(f\right)\cdot \underset{F\{i_{d}\left[i\right]\}}{\underbrace{I_{d}\left(f\right)}}\right\},$$

where $v_{c0}$ is the operating voltage of the circuit model, $F^{-1}$ is the inverse discrete Fourier transform (IDFT), F shows the discrete Fourier transform (DFT), and $\Delta v_{c}\left[i\right]$ denotes the small-signal voltage of the circuit model [47]. The output voltages of the circuit module are given in Figure 10.

### 5.5. Ranging Module

The ranging algorithm takes the voltage signal $v_{c}\left[i\right]$ from the circuit module as its input. Then, it calculates the target range and the signal intensity for each scan point. The range is given in meters while the intensity is mapped linearly to an arbitrary integer scale from 0 to 4096 as used in the Cube 1 products.

**Figure 11:**
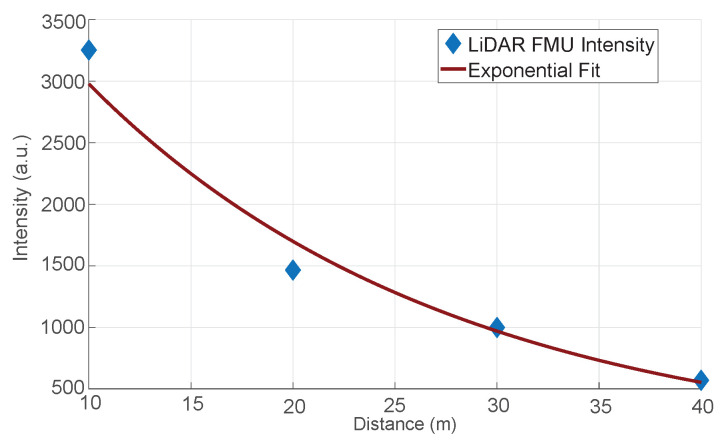
The output of the ranging module for 5% reflective point scatter targets.

The algorithm applies several threshold levels to distinguish between internal reflection, noise, and target peaks. The target range is determined based on the relative position of the target peaks to the internal reflection, while the signal intensity is calculated from the peak voltage levels. The output of the ranging module is given in Figure 11.

## 6. Results

The model’s accuracy presented in this paper is authenticate on two interfaces: time-domain and point cloud. We used single-point scatter to validate the model in the time domain.

### 6.1. Validation of the Model on Time Domain

The primary reason to verify the LiDAR model on the time domain is that the link budget, detector, and circuit modules are working as intended. Furthermore, comparing the time domain signals (TDS) establishes the association between measured and modeled noise and amplitude levels because it is difficult to compare the simulated and measured noise at the point cloud level.

**Figure 12:**
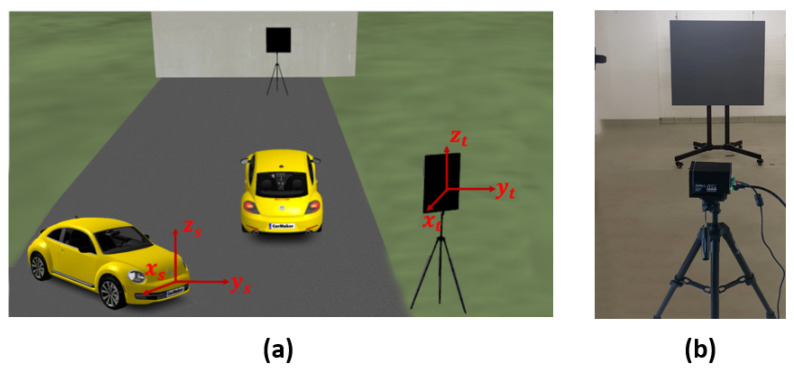
(**a**) Static simulation scene to validate the time domain and point cloud data. (**b**) Real setup to validate the time domain and point cloud data. The 10% reflective target was placed in front of the sensor at different distances. The coordinates of the actual and simulated sensor and target are the same. The ground truth distance $d_{GT}$ is calculated from the sensor origin to the target center.

**Figure 13:**
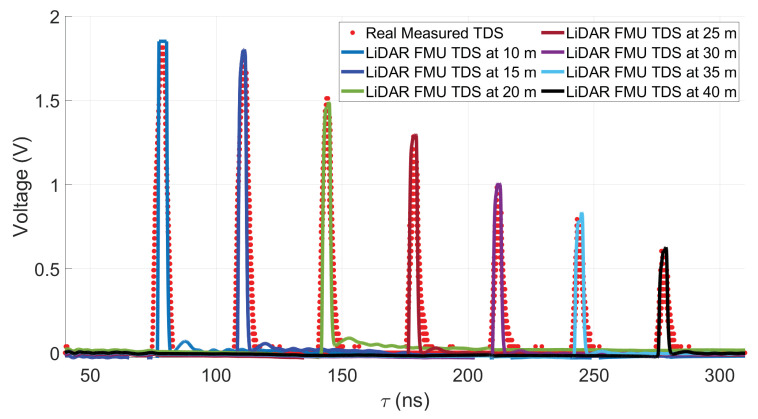
LiDAR FMU and real measured TDS comparison. The target peaks and noise levels match well. Furthermore, the LiDAR FMU model provides the same results in AURELION from dSPACE for the time domain signals. We used the *osi3::GroundTruth* interface to get the target’s position in the virtual environment.

**Figure 14:**
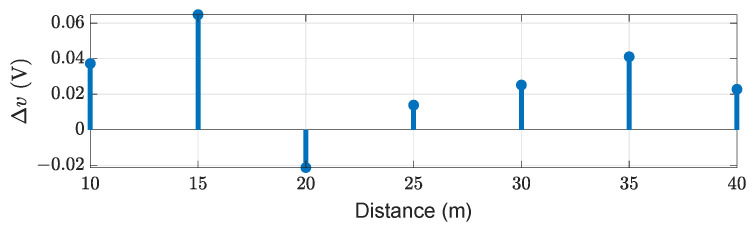
The voltages difference Δv of simulated and measured target peaks.

A 10% diffuse reflective Lambert plate is placed at the distance of 10 m, 15 m, 20 m, 25 m, 30 m, 35 m, and 40 m in front of the sensor, as shown in Figure 12. To verify the model on the time domain level, only single-point scatter is considered from the surface of the target. The comparison of the simulated and real measured TDS and their amplitude differences Δv are shown in Figure 13 and Figure 14, respectively. The amplitude difference Δv can be written as

(11) $$\Delta v=v_{sim}-v_{real},$$

where $v_{sim}$ is LiDAR FMU TDS, and $v_{real}$ denotes the measured TDS of the LiDAR sensor.

It can be seen that object’s peaks, shape, and noise level match quite well. But the difference in amplitude Δv can be observed at different distances, as given in Figure 14. This is because we use the small-signal transfer function, not an analog circuit model, to prevent computational burden. To quantify the amplitude difference Δv, we use the mean absolute percentage error (MAPE ) metric

(12) $$MAPE=\frac{1}{n}\sum_{i=1}^{n}|\frac{y_{i}-x_{i}}{y_{i}}|,$$

where $y_{i}$ is the simulated value, the measured value is denoted by $x_{i}$, and *n* shows the total number of data points [56]. The MAPE of voltages is 1.7%.

**Figure 15:**
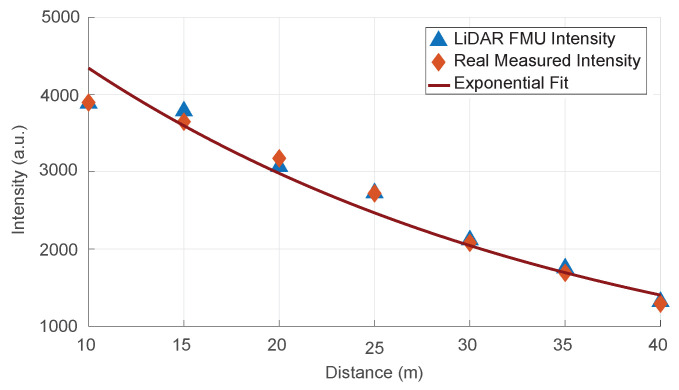
The validation of the ranging module. The simulated and measured intensities values show good agreement.

Afterward, we validated the ranging model by comparing the intensities value shown in Figure 15. Again, the result shows good agreement between the simulated and measured data. Here, it should be noted that the voltage mismatch is directly proportional to the intensities discrepancy because the voltages are mapped linearly to the arbitrary integer intensity scale.

### 6.2. Validation of the Model on Point Cloud Level

To validate the sensor model on the point cloud level, we took the lab tests in ideal conditions and proving ground measurements in real environmental conditions.

#### 6.2.1. Lab Test

In the next step, the model is analyzed at the point cloud level, and for this purpose, the same test setup is used, as shown in Figure 12. Now, all the reflections from the Lambertian plates during the scan are considered. The performance of the LiDAR sensor model significantly depends upon its fidelity level and virtual environmental modeling, including the target’s surface reflective properties. However, as mentioned earlier, no metrics or KPIs are available to verify the sensor model accuracy at the point cloud level. Therefore, we define three KPIs based on expert knowledge to confirm whether the model is ready for ADAS testing, as follows.

(1) The number of received points $N_{points}$ from the surface of the simulated and real objects of interest.
(2) The comparison between the mean intensity $I_{mean}$ values of received reflections from the surface of the simulated and real targets.
(3) The distance error $d_{error}$ of point clouds obtained from the actual and virtual objects should not be more than the range accuracy of the real sensor, which is 2 cm in this case.

The number of received points $N_{points}$ is an important KPI. Because neural networks and deep learning algorithms are applied to 3D LiDAR point clouds for object recognition, segmentation, and classification. If the number of LiDAR points $N_{points}$ received from the simulated and measured objects is different. It will influence the performance of object-recognition algorithms and the ADAS. This KPI depends on the simulated and real scan pattern’s similarity. For this paper, the LiDAR sensor model and LiDAR sensor use the same scan pattern shown in Figure 6. In the author’s opinion, this is an important KPI to be considered for the accuracy verification of the model.

The intensity values of received reflections in simulation and actual measurement are also considered for the environmental modeling and sensor model verification. However, if the reflectivity of the modeled object is not the same as in the real world, mean intensity values $I_{mean}$ and the number of received reflections $N_{points}$ will not match. Therefore, this KPI is also essential to obtain a realistic output.

Furthermore, the distance error $d_{error}$ of the point clouds received from the simulated and measured object of interest should not be more than the range accuracy of the real sensor. We have

(13) $$d_{error}=d_{GT}-d_{mean,sim/meas},$$

where ground truth distance is denoted by $d_{GT}$, $d_{mean,sim}$, and $d_{mean,meas}$ are the mean distance of reflections received from the surface of simulated and the real object of interest. The ground truth distance $d_{GT}$ is calculated from the sensor’s origin to the target’s center, and it can be written as

(14) $$d_{GT}=\sqrt{{\left(x_{t}-xs\right)}^{2}+{\left(y_{t}-y_{s}\right)}^{2}+{\left(z_{t}-z_{s}\right)}^{2}},$$

where the target’s *x*, *y*, and *z* coordinates are denoted by subscript *t* and sensors by *s* [57]. OSI ground truth interface *osi3::GroundTruth* is used to retrieve the sensor origin and target center position in 3D coordinates.

**Figure 16:**
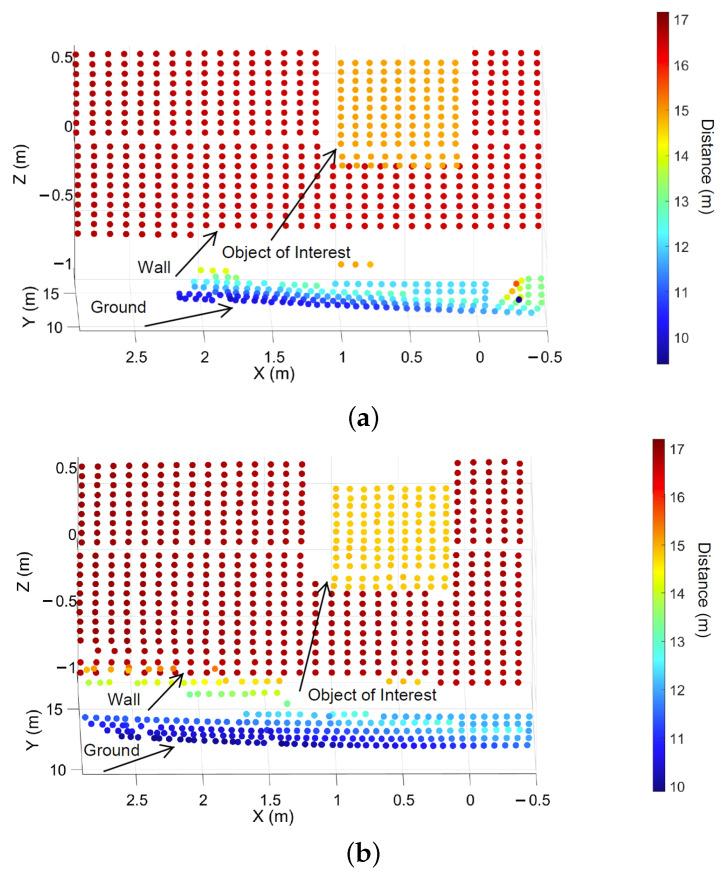
Exemplary visualization of the Cartesian point clouds received from all the objects in the FoV of LiDAR FMU and real sensor. (**a**) The LiDAR FMU 3D Cartesian point clouds. (**b**) The 3D point clouds of real sensors. It should be noted that the modeled and actual objects’ material properties are different except for the Lambertian target. That’s why the number of points $N_{points}$ received from the ground and walls are different in simulation and actual measurement.

**Figure 17:**
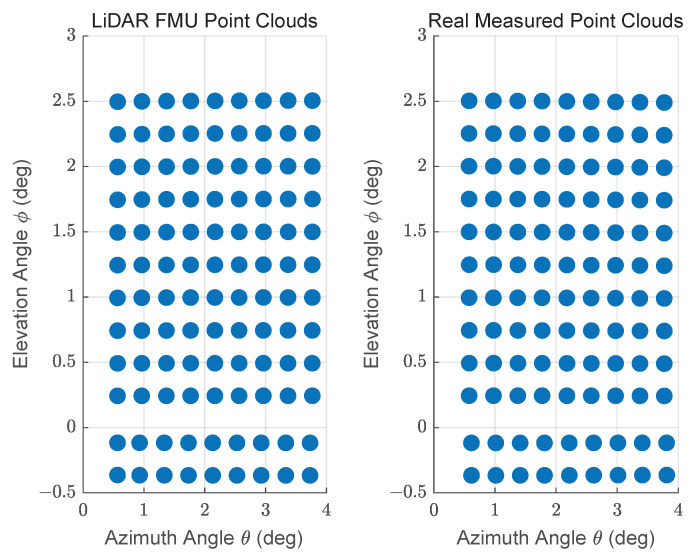
Visualization in spherical coordinates of points obtained from the actual and simulated Lambertian plate placed at 15 m. The horizontal spacing between the simulated and measured points is $\theta =0.4^{\circ }$ and vertical spacing is $\phi =0.25^{\circ }$.

The exemplary 3D point clouds of LiDAR FMU and real measurement are given in Figure 16a,b, respectively. Figure 17 shows the simulated and real measured spherical point clouds obtained from the Lambertian plates. The horizontal θ and vertical ϕ spacing of simulated and real measured point clouds are $0.4^{\circ }$ and $0.25^{\circ }$, respectively. That shows the horizontal and vertical spacing of points in simulated and real scan patterns are the same.

**Figure 18:**
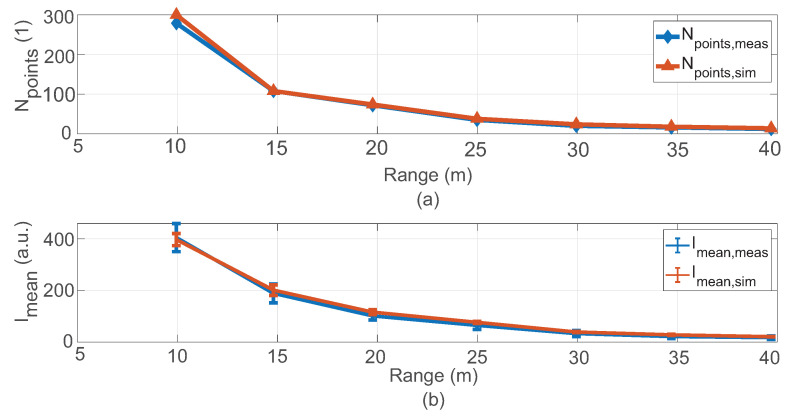
(**a**) The number of received points from the object of interest in simulation and real measurement is approximately the same at all distance values. However, a slight mismatch in the number of reflections can be observed because it is impossible to replicate the 100% real-world conditions in the simulation, for instance, ambient light. (**b**) The mean intensity $I_{mean}$ values show good agreement. It can also be observed that the standard deviation of real measured intensity values is higher than the simulated intensity values because the ambient light condition influences the real measured intensity values.

Figure 18 compares the number of received points $N_{points}$, and mean intensity values $I_{mean}$. The number of received points $N_{points}$ and mean intensity $I_{mean}$ from the object of interest in the simulation and real measurements show good agreement. However, a slight mismatch between these quantities can be observed. The possible reasons for the deviation are the ambient light that is not constant over the entire measurement field. We used the MAPE metric as given in Equation (12) to quantify the difference between the number of received points $N_{points}$ and mean intensity $I_{mean}$. The results are given in Table 2.

**Figure 19:**
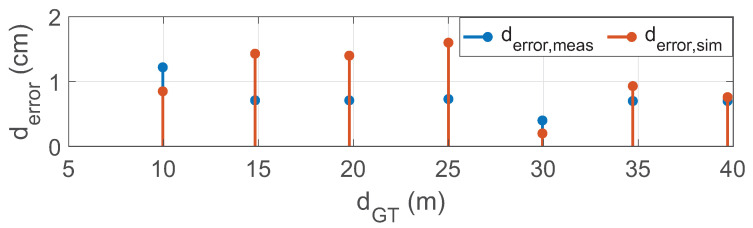
The distance error is below the range accuracy, that is ±2 cm.

The distance error $d_{error}$ is shown in Figure 19, and it is below the range accuracy of the real sensor, which is 2 cm in this case. These results confirm that the scan pattern and modeled object reflective properties are the same as the real sensor and object. Furthermore, the sensor model provides realistic output.

#### 6.2.2. Proving Ground Tests

**Figure 20:**
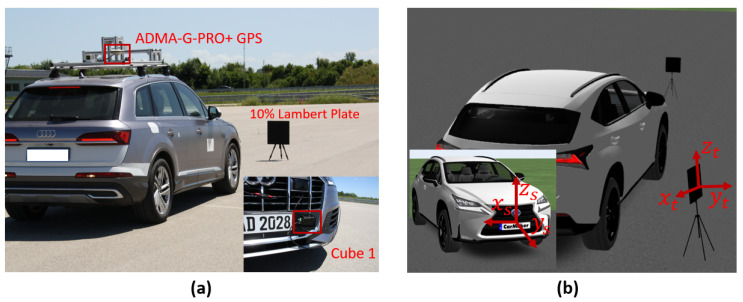
(**a**) The test vehicle was equipped with a LiDAR sensor and global positioning system (GPS). The GPS ADMA-G-PRO+ from Genesys Inc. is used as the reference sensor with a range accuracy of 0.1 m. The size of the 10% reflective plate is 0.5 × 0.5. (**b**) The static simulation scene. The ground truth distance $d_{GT}$ is calculated from the sensor reference point to the center of the plate target in simulation and real measurement by using Equation (14).

**Figure 21:**
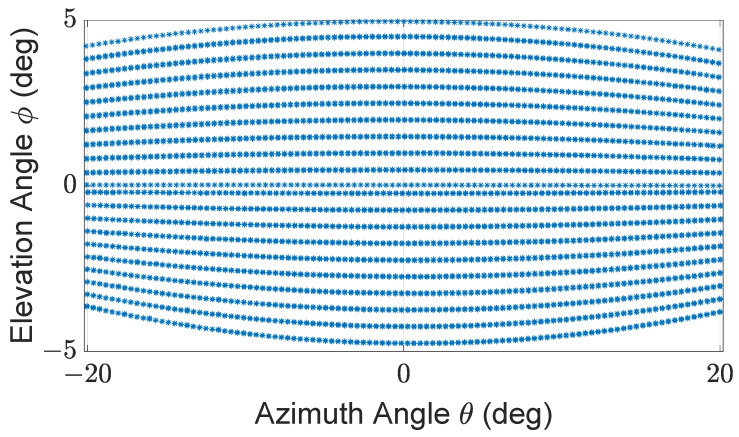
Specification of scan pattern used by the real and virtual LiDAR sensors for proving ground tests: $42^{\circ }$ horizontal and $10^{\circ }$ vertical FoV, 40 scan lines, frame mode only up, $0.4^{\circ }$ horizontal angle spacing, frame rate 13.3 Hz, maximum detection range is 250 m, and minimum detection range is 4.80 m.

We have conducted static tests at the FAKT-motion GmbH proving ground in Benningen to record the real data in daylight. The intensity of daylight was 8 klux. A 10% reflective Lambertian plate is placed at the distance of 5 m, 10 m, 15 m, 20 m, 25 m, 30 m, 35 m, and 40 m in front of the Cube 1 mounted on the test vehicle as shown in Figure 20. The scan pattern used for this measurement campaign is shown in Figure 21.

**Figure 22:**
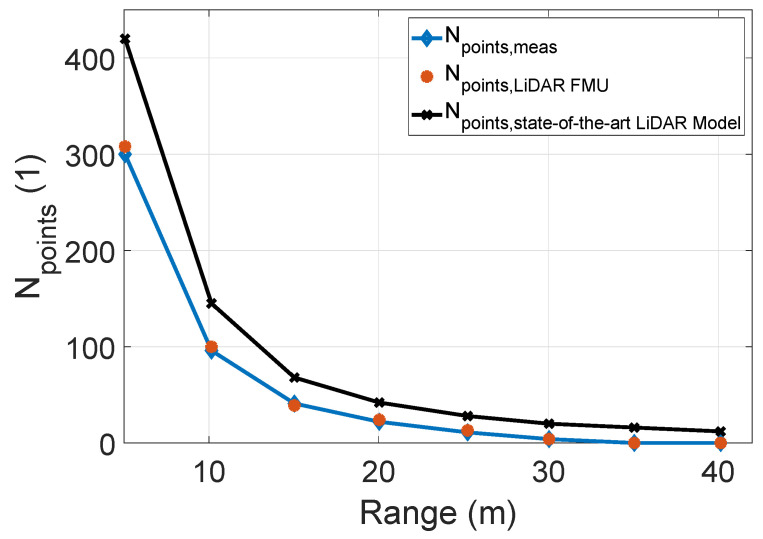
The comparison between the number of received points $N_{points}$ obtained from the simulated and real 10% Lambertian plate. The actual measured and LiDAR FMU received point cloud $N_{points}$ are similar. However, the number of points $N_{points}$ yielded by the state-of-the-art LiDAR sensor model are higher. The MAPE for the number of received $N_{points}$ of LiDAR FMU is 9.6% and 48.4% for the state-of-the-art LiDAR sensor model up to 30 m.

**Figure 23:**
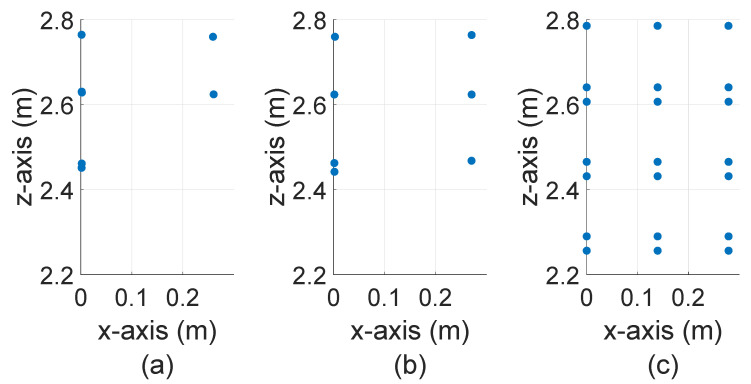
The exemplary point clouds provided by real and virtual LiDAR sensor models for 0.5 m × 0.5 m Lambertian plate placed at 30 m. (**a**) Real measured point clouds (**b**) LiDAR FMU point clouds (**c**) State-of-the-art LiDAR sensor model. The real and LiDAR FMU points are noisy and dispersed. However, the state-of-the-art LiDAR model points are ideal and aligned.

Figure 22 compares the number of received points $N_{points}$ for the real and simulated object of interest. It should be noted the sensor model provided by the CarMaker also uses the same scan pattern, as shown in Figure 21. The real sensor and LiDAR FMU model can detect the object of interest up to the 30 m. However, the exact dimension of the target cannot be estimated from the real and LiDAR FMU point clouds, as shown in Figure 23. This is because the background noise due to the sunlight, detector shot noise, and thermal noise of electronic circuitry masks the weak reflections received from the Lambertian target. Consequently, the peak detection algorithm of real and LiDAR FMU models cannot distinguish between the noise and target peaks. It should be noted LiDAR FMU model uses the same peak detection algorithm and detection thresholds as Cube 1. On the other hand, a state-of-the-art sensor model provided by commercial software can detect the target till 40 m, and the dimension of the target can be estimated easily from the yielded point clouds. This is the case because the LiDAR models provided by the commercial software are generic and parameterizable. These sensor models do not consider the complete signal processing toolchain of specific LiDAR hardware and related sensor imperfections. Instead, they allow the user to integrate the sensor-specific scan pattern, obtain the ideal point clouds, and apply the signal processing steps and imperfection of the LiDAR sensor to get simulation results close to the specific LiDAR sensor in post-processing.

The authors believe the sensor models provided by the commercial and open source tool vendors can be used for the ADAS testing that requires ideal or medium fidelity point clouds. However, in use cases where a high-fidelity LiDAR model’s output is required, the scan pattern, complete signal processing toolchain, and sensor-specific imperfections of real LiDAR sensor, as mentioned in Section 1 need to be considered.

**Figure 24:**
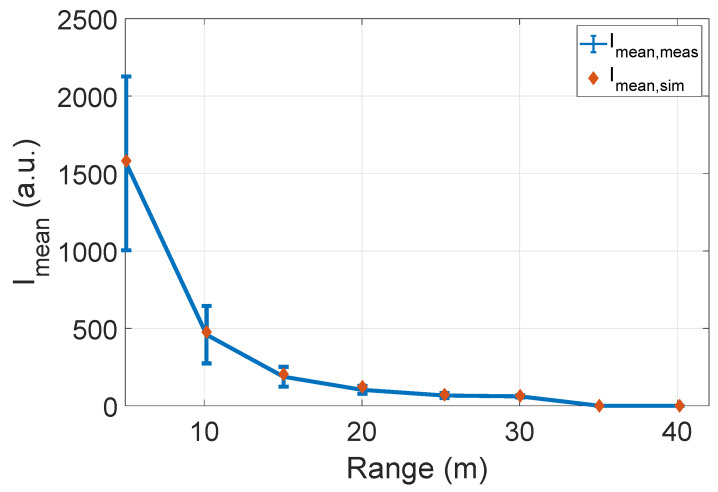
The comparison of measured and simulated mean intensity $I_{mean}$ values. The mean intensity $I_{mean}$ values show good agreement. The MAPE for the mean intensity $I_{mean}$ is 11.1%. It should be noted that comparing intensity values of the real measurement and state-of-the-art sensor model is impossible because their units are different.

**Figure 25:**
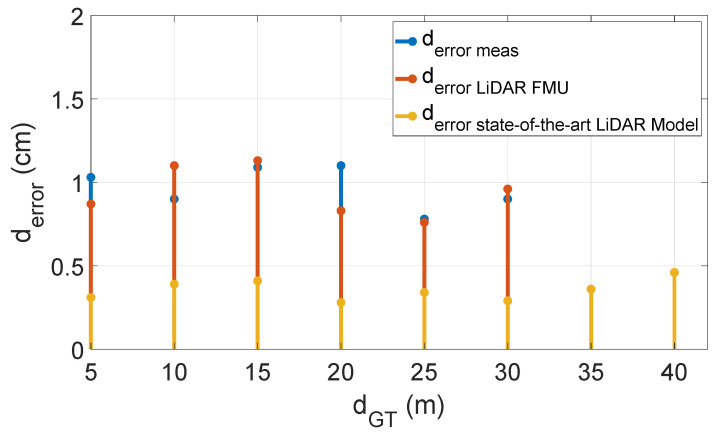
The distance error $d_{error}$ of real and virtual sensors is below the range accuracy of the real sensor ±2 cm.

Figure 24 compares the mean intensity $I_{mean}$ of Cube 1 and LiDAR FMU model. The mean intensity $I_{mean}$ values received from the object of interest in simulation and real measurement are similar. We used Equation (12) to quantify the difference between the simulated and real measured values. The MAPE for the mean intensity $I_{mean}$ is 11.1%, and it is greater than the MAPE of lab tests mean intensity $I_{mean}$ values as given in Section 6.2.1. Although we have modeled the daylight intensity in the LiDAR FMU model, it is still challenging to model 100% environmental conditions in the simulation. The increase in the MAPE for mean intensity $I_{mean}$ values is due to environmental losses. Furthermore, it is impossible to compare state-of-the-art LiDAR sensor model intensity with the real measurement because their signal processing steps to calculate the intensity are not the same, which is why their units are different. The real measured point cloud intensity is in arbitrary units (a.u.), and state-of-the-art sensor model intensity is in Watts. Figure 25 shows the comparison of distance error $d_{error}$ of the real and virtual sensors. The distance error $d_{error}$ of the state-of-the-art LiDAR model is less than 0.5 cm. This is because this sensor model provides the ideal point clouds, and its mean distance $d_{mean}$ is closer to the ground truth distance $d_{GT}$. However, the real LiDAR sensor and LiDAR FMU point clouds are noisy and dispersed, which is why their distance error $d_{error}$ is more than 1 cm.

## 7. Conclusions

In this work, we have introduced a process to develop a tool-independent, high-fidelity LiDAR sensor model by using FMI and OSI standardized interfaces. The model was integrated successfully into the virtual environment of CarMaker and AURELION from dSPACE to show its exchangeability. Moreover, the LiDAR FMU model provides the same results regardless of the tool used. The developed LiDAR sensor model includes the complete signal processing steps of the real LiDAR sensor and considers the sensor-specific imperfections, including optical losses, inherent detector effects, effects generated by the electrical amplification, and noise produced by the sunlight to output the realistic data. The virtual LiDAR sensor outputs the time domain and point cloud data. The real and simulated time domain results comparison show that simulated and measured signals’ peak shape, noise, and amplitude levels match well. The MAPE of amplitude is 1.7%. Furthermore, KPIs are defined to authenticate the simulated and measured point clouds. The presented lab test results demonstrate that the MAPE is 8.5% and 9.3% for the number of points $N_{points}$ and mean intensity values $I_{mean}$ obtained from the simulated and real Lambertian plates. Moreover, the distance error $d_{error}$ is below 2 cm. In addition, the static tests were performed at the proving ground on a sunny day to record the real data. Real measurement results are compared with the state-of-the-art LiDAR model provided by the commercial and the proposed LiDAR model to show the presented model fidelity. The results show that although the state-of-the-art LiDAR model uses a similar scan pattern as the real sensor, it cannot exhibit the same results as a real sensor. This is because it does not include the complete signal processing steps of the real sensor and related imperfections. Such models are useful for testing the ADAS in use cases where low and medium-fidelity LiDAR point clouds are sufficient. The scan pattern, complete signal processing steps, and sensor-specific imperfections must be considered when high-fidelity output is required. It is also concluded that the material properties and reflectivity of the modeled and actual object of interest should be the same; otherwise, simulation and actual results will not match.

## 8. Outlook

The model will be further validated in the next steps as per the ASTM E3125-17 standard. Moreover, the model fidelity will be validated using the different state-of-the-art metrics available. Furthermore, rain and fog effects on the performance of automotive LiDAR sensors will be modeled and validated. 

## Figures and Tables

**Table 1:** Overview of the state-of-the-art LiDAR sensor model working principles and validation approaches.

Authors	Model Type	Input of Model	Output of Model	Covered Effects	Validation Approach
Hanke et al. [23]	Ideal/low-fidelity	Object list	Object list	FoV and object occlusion	N/A
Stolz & Nestlinger [24]	Ideal/low-fidelity	Object list	Object list	FoV and object occlusion	N/A
Muckenhuber et al. [26]	Phenomenological/ low-fidelity	Object list	Object list	FoV, object class definition, occlusion, probability of false positive and false negative detections	Simulation result
Linnhoff et al. [27]	Phenomenological/ low-fidelity	Object list	Object list	Partial occlusion of objects, limitation of angular view, and decrease in the effective range due to atmospheric attenuation	Simulation result comparison with ray tracing model at object level
Hirsenkorn et al. [25]	Phenomenological/ low-fidelity	Object list	Object list	Ranging errors, latency, false-positive, and occlusion	Simulation result
Zhao et al. [28]	Phenomenological/ low-fidelity	Object list	Object list or point clouds	Occlusion, FoV and beam divergence	Simulation result
Li et al. [29]	Physical/ medium-fidelity	Object list	Object list or point clouds	Occlusion, FoV and beam divergence	Simulation result
Philipp et al. [31]	Physical/ medium-fidelity	Ray-casting	Point clouds & object list	Beam divergence, SNR, detection threshold, and material surface properties	Qualitative compar- ison with real and re- ference measuremen- ts at the object list le- vel for one dynamic scenario
Gschwandtner et al. [32]	Physical/ medium-fidelity	Ray-casting	Point clouds	Sensor noise, materials physical properties, and FSPL	Simulation results
Goodin et al. [33]	Physical/ medium-fidelity	Ray-casting	Point clouds	Beam divergence and a Gaussian beam profile	Simulation results
Bechtold & Höfle [34]	Physical/ medium-fidelity	Ray-casting	Point clouds	Beam divergence, atmospheric attenuation, scanner efficiency, and material surface properties	Simulation results
Hanke et al. [35]	Physical/ medium-fidelity	Ray-tracing	Point clouds	Beam divergence, material surface properties, detection threshold, noise effects, and atmospheric attenuation	Qualitative comparis- on of synthetic and re- al data at point cloud level for one dynamic scenario
Li et al. [29]	Physical/ medium-fidelity	Ray-tracing	Point clouds	Beam divergence, power loss due to rain, fog, snow, and haze	Simulation results for one static and one dy- namic scenario
Zhao et al. [28]	Physical/ medium-fidelity	Ray-tracing	Point clouds	False alarm due to the backscattering from water droplets	Qualitative comparis- on with measurement
CARLA [37]	Physical/ medium-fidelity	Ray-casting	Point clouds	signal attenuation, noise the drop-off in number of point clouds loss due to external perturbations	N/A
CarMaker [20]	Physical/ medium-fidelity	Ray-tracing	Point clouds	Noise, the drop-off in intensity, and the number of point clouds due to atmospheric attenuation	N/A
DYNA4 [38]	Physical/ medium-fidelity	Ray-casting	Point clouds	Physical effects, the material surface reflectivity and ray angle of incidence	N/A
VTD [40]	Physical/ medium-fidelity	Ray-tracing	Point clouds	Material properties	N/A
AURELION [39]	Physical/ medium-fidelity	Ray-tracing	Point clouds	Material surface reflectivity, sensor noise, atmospheric attenuation, and fast motion scan effect	N/A
Haider et al. (proposed model)	Physical/ high-fidelity	Ray-tracing	Time domain & point clouds	Material surface reflectivity, beam divergence, FSPL daylight, daylight filter, internal reflection of detector saturation of detector from bright targets, detector shot noise and dark count rate, and detection threshold	Qualitative comparison of simulation and real measurement at time do- main and point cloud level

**Table 2:** The MAPE for the number of points $N_{points}$ and mean intensity $I_{mean}$.

Parameter	MAPE
Number of received points $N_{points}$	8.5%
Mean Intensity $I_{mean}$	9.3%

## Data Availability

Not applicable.

## Abbreviations

The following abbreviations are used in this manuscript:

ADAS	Advanced Driver-Assistance System
LiDAR	Light Detection And Ranging
RADAR	Radio Detection And Ranging
FMU	Functional Mock-Up Unit (FMU)
OSI	Open Simulation Interface
FMI	Functional Mock-Up Interface (FMI)
MAPE	Mean Absolute Percentage Error
ABS	Anti-Lock Braking System
ACC	Adaptive Cruise Control
ESC	Electronic Stability Control
LDW	Lane Departure Warning
PA	Parking Assistant
TSR	Traffic-Sign Recognition
MiL	Model-in-the-Loop
HiL	Hardware-in-the-Loop
SiL	Software-in-the-Loop
IP	Intellectual Property
KPIs	Key Performance Indicators
OEMs	Original Equipment Manufacturers
FoV	Field of View
RTDT	Round-Trip Delay Time
ToF	Time of Flight
HAD	Highly Automated Driving
RSI	Raw Signal Interface
OSMP	OSI Sensor Model Packaging
SNR	Signal-to-Noise Ratio
FSPL	Free Space Path Losses
BRDF	Bidirectional Reflectance Distribution Function
SiPM	Silicon Photomultipliers
APD	Avalanche Photodiode
SPAD	Single-Photon Avalanche diode
FX Engine	Effect Engine
MEMS	Microelectromechanical Mirrors
IDFT	Inverse discrete Fourier Transform
DFT	Discrete Fourier Transform
TDS	Time Domain Signals
